# Determination of optimum irrigation strategies and effect of drip irrigation system on growth and water use efficiency of pear jujube in Loess Hilly region of northern Shaanxi

**DOI:** 10.1371/journal.pone.0221925

**Published:** 2019-08-29

**Authors:** Shenglan Ye, Jichang Han, Tiancheng Liu

**Affiliations:** 1 Shaanxi Provincial Land Engineering Construction Group Co., Ltd., Shaanxi, Xi’an, China; 2 Institute of Land Engineering and Technology, Shaanxi Provincial Land Engineering Construction Group Co., Ltd., Shaanxi, Xi’an, China; Huazhong Agriculture University, CHINA

## Abstract

The aim of this study is to explore suitable drip irrigation system on the water saving and high yield of pear-jujube from 2009 to 2012 years in the mountain of northern Shaanxi. The treatments consisted of combinations of 5 drip irrigation systems (DP). The irrigation quota of DP-1, DP-2 and DP-3 treatment was 100 m^3^ hm^-2^, 135 m^3^ hm^-2^ and 180 m^3^ hm^-2^, respectively, irrigated 4 times. The irrigation quota of DP-4 and DP-5 treatment was 135 m^3^ hm^-2^ and irrigated 3 and 2times, respectively; and with no irrigation as the control (C). Results indicated that bearing branch length of jujube, fruit set and yield of different drip irrigation system are significantly better than C (*P*<0.05). Bearing branch length and yield of DP-3 treatment are reached maximum in 2012, which are 22.0 cm and 16772.8 m^3^ hm^-2^. And they are increased by 47.7% and 13.2% compared with C, respectively. In addition, the water consumption of different irrigation treatment increases along with the increasing of irrigation amount. And the DP-3 treatment is the highest in different years. The water use efficiency of pear-jujube of low irrigation quota is better than the high irrigation quota. Water use efficiency of 135 m^3^ hm^-2^ and irrigated 2 times treatment is the best, which is 1.92 m^3^ hm^-2^. Considering the lack of high annual precipitation, we conclude that DP-5 treatment was the best drip irrigation system in the mountain of northern Shaanxi.

## Introduction

*Zizyphus jujuba* Mill. is a genus of Zizyphus Mill. [1]. Jujube trees are planted in the hilly area of the Loess Plateau. It can not only green barren hills, resist natural disasters, maintain water and soil, but also improve farmers' economic benefits. The research jujube area is located in the northern part of the Loess Plateau where it was little rainfall with an average annual rainfall of 451.6 mm; and the spatial and temporal distribution is unbalanced. The rainfall is mainly concentrated in July to September, with severe spring drought, frequent drought and autumn drought. Under such conditions of drought and little rain, water resources are extremely precious. Planting jujube is limited by the shortage of water resources, such as the low survival rate of jujube planting, the small amount of growth in the early stage, concentrated flowering, and the delayed fruits. The economic benefits are extremely low, which seriously affects the enthusiasm of farmers for planting jujube; in turn it affects the improvement of the ecological environment in northern Shaanxi.

At present, drip irrigation technology is a modern water-saving system with more precise water quantity control and higher water use efficiency. Irrigation has a regulating effect on the environmental conditions such as water, fertilizer, gas and heat, and the soil water and heat distribution have a significant effect on crop growth and yield [2–3]. This technology is commonly used in the arid and semi-arid areas of northern China, especially in the production of jujube in northern Shaanxi [4]. In agricultural production, attention should be given to irrigation techniques and water use efficiency. Studies have shown that water use efficiency is an important indicator of water-saving irrigation; a key indicator for measuring the relationship between crop yield and water use efficiency [5]. And water use efficiency expresses the relationship between plant water consumption and productivity. Under drought conditions, water use efficiency is higher; but under watery conditions, plant water use efficiency is lower [6]. Ma et al. [7] showed that drip irrigation can quickly moisten the surface soil, which is conducive to the absorption and utilization of water by crops. The research revealed significant linear relationships among the irrigation water, water consumption and total number of flowers per plant [8]. Irrigation system experiments on the melon in the semi-arid Ankara region of Turkey have indicated that irrigation during melon ripening does not significantly affect fruit yield and sugar content [9]. Che et al. [10] study different irrigation methods (root irrigation, drip irrigation, ground irrigation, etc.) and different irrigation quotas for pear jujube; the Yongquan root irrigation and drip irrigation significantly increased jujube yield compared with flood irrigation. The yield increases with the irrigation quota and then decreases, but the water use efficiency decreases with the increase of irrigation quota. Some studies have found that drip irrigation is more effective than flood irrigation to increase crop yield and water use efficiency, and crop yield increases significantly as irrigation increases [11–12]. Abrisqueta et al. [13] found that continuous regulated deficit irrigation of peach trees can inhibit the growth of roots and improve water use efficiency. Intrigliolo et al. [14] indicated that under the water-saving irrigation, the yield and quality of the grapes were not affected. The above studies show that water-saving irrigation has been widely applied to the irrigation of fruit tree. However, there are regional differences, crop species differences and climate differences; especially the research on irrigation system of pear jujube in semi-arid areas of northern Shaanxi is rarely reported. The eight-year-old dwarf and densely planted pear jujube in northern Shaanxi was used as a study material. The effects of different irrigation quotas and irrigation times on the growth and water use efficiency of pear jujube was compared for consecutive 4 years. Explore the optimal drip irrigation system suitable for pear jujube forest in the Loess Plateau of northern Shaanxi; it can provide a theoretical and empirical basis for guiding the high water-saving and high yield of local pear jujube. It is of great significance to realize water-saving irrigation production in northern Shaanxi.

## Materials and methods

### Overview of the study site

The study was carried out in Mengzi Village, Mizhi County, Yulin City where is pear jujube planting base. The study tree was 8 years old with uniform growth and good growth. The landform of study area belongs to the typical hilly and gully region of the Loess Plateau. The soil was dominated by Lossiah soil. Bulk density was 1.21 g cm^-3^; and soil fertility is low [15]. The climate is typical of the warm temperate and semi-arid climates. The temperature difference between day and night was large. It was suitable for fruit tree growth. Average annual precipitation is 451.6 mm. The average temperature is 8.8°C. The frost-free period is 160 days. The particle gradation composition of soil in the study area is shown in Table 1.

**Table 1:** Study soil particle gradation composition table.

**Particle size (mm)**	<0.002	0.002–0.02	0.02–0.20	0.2–2.0
**Particle amount (%)**	17.55	42.59	38.86	1.0

### Study designs

The drip irrigation study was conducted during the 2009 to 2012 years. The study site is a horizontal terrace on the slope. The soil water and fertilizer and other factors along the contour line are basically the same. The study trees are arranged along the contour line. The irrigation amount of pear jujube only needs to satisfy the soil moisture requirement of 1/4 root schedule; the irrigation amount per plant is the water consumption of pear jujube in 2008 [16]. The soil moisture content is restricted to 80% and 60% of the field capacity. The soil depth is 80 cm and the wetted area is 6 m^2^. The irrigation quota of single pear jujube was calculated by the formula to be 135 m^3^ hm^-2^. The formula is: irrigation quota = planned wet depth × planned wet area × (field water holding rate-soil moisture content [17]. According to the characteristics of large rainfall from August to September in the Loess Plateau of northern Shaanxi and the water demand of jujube trees, the irrigation quota is set to 180, 135, 100 m^3^ hm^-2^. The irrigation time are mainly in the sprouting stage, flowering fruit stage and expansion stage. The times of irrigations was set to 4, 3, and 2. Individual plant is one treatment, and set 5 replicates. The study area is 300 m^2^. The planting density is 2 m × 3 m. The study plan is shown in Table 2.

**Table 2:** Study plan.

Treatment	Irrigation quota (m^3^ hm^-2^)	Irrigation times	Irrigation time			
			Budding leaf period	Flowering fruit setting period	expansion period	maturity period
**DP-1**	100	4	04/20	05/17, 06/12	07/16	0
**DP-2**	135	4	04/20	05/17, 06/12	07/16	0
**DP-3**	180	4	04/20	05/17, 06/12	07/16	0
**DP-4**	135	3	04/20	05/17, 06/12	0	0
**DP-5**	135	2	04/20	05/17	0	0
**C**	0					

### Measurement indicators and methods

Fruit setting rate: Take the fixed eight-point bearing branch in different directions of jujube trees. Record the number of flowers and fruits in the flowering and fruiting stages, respectively, and the average value was taken. Fruit set rate = number of fruits/number of flowers × 100%.

Yield: After the fruit is mature, the single plant single-receipt method is adopted. Every time the fruit is picked, it is recorded until the fruit picking is completed. Calculate individual yield and convert to total yield.

Soil moisture content: The soil moisture content is determined by the drying method. One layer per 20 cm, the measurement range is 0 to 100 cm.

Water consumption of pear jujube (*ET*): The water balance method in the direct method is used to calculate the water consumption of jujube trees.

Jujube crop coefficient (*K*c): It is the ratio of actual crop water consumption to reference crop water demand. The reference crop water requirement is calculated using the more common Penman-Monteith formula at home and abroad [18]. The required meteorological data was obtained from the Mizhi County Weather Station.

Water use efficiency (*WUE*): *WUE* (kg hm^-2^ mm^-1^) = *Y*/*ET*. Y is the yield of pear jujube, kg hm^-2^. ET is the water consumption (mm) of jujube trees.

The study data was compiled using Microsoft Excel 2010, and a significant difference study was performed using SPSS 17.0.

## Results

### Influence of different irrigation system on bearing branch length of pear jujube

**Fig 1:**
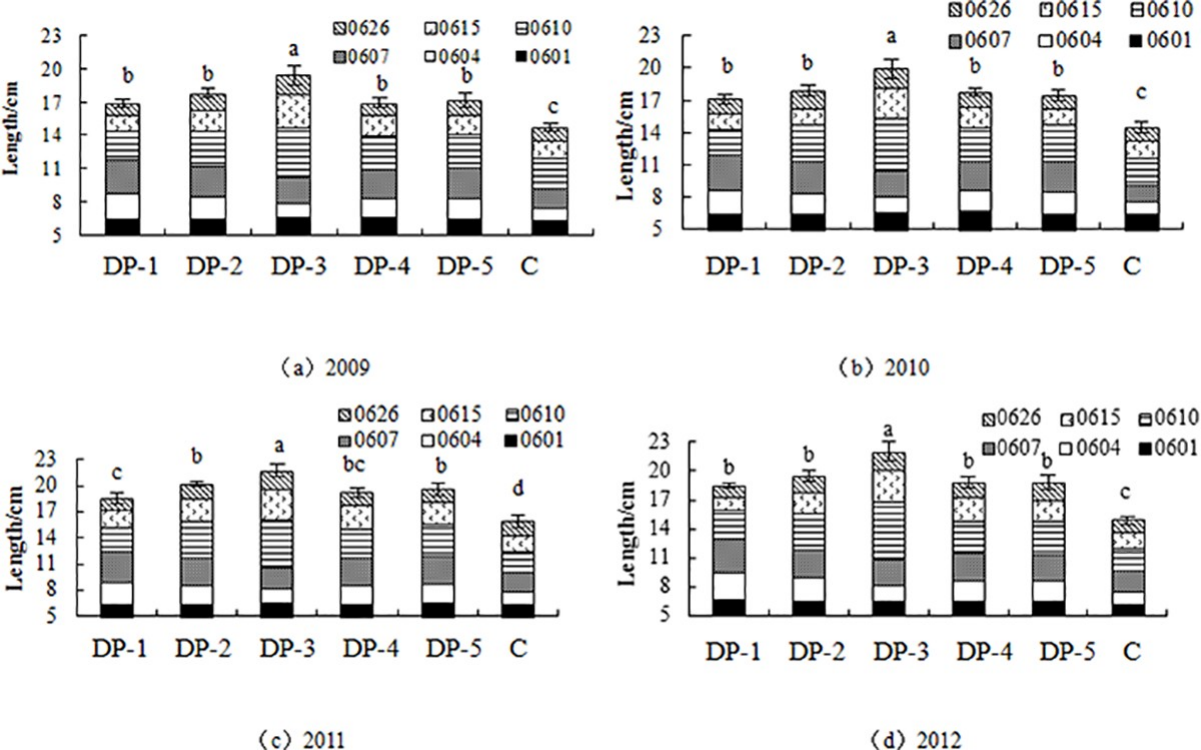
Bearing branch length in different irrigation system on a), b) c), d) from 2009 to 2012 (Mean ± SD) Means denoted by different letters under the same. As level indicate significant difference at 0.5% level of significance. DP-1: irrigation quota of 100 m^3^ hm^-2^ and irrigation 4 times; DP-2: irrigation quota of 135 m^3^ hm^-2^ and irrigation 4 times;DP-3: irrigation quota of 180 m^3^ hm^-2^ and irrigation 4 times;DP-4: irrigation quota of 135 m^3^ hm^-2^ and irrigation 3 times;DP-5: irrigation quota of 135 m^3^ hm^-2^ and irrigation 2 times; C: no irrigation.

Different irrigation systems significantly increase the growth rate of jujube bearing branch; the growth rate of bearing branch reaches the maximum on June 10 (Fig 1A–1D). With the increase of irrigation amount, the growth rate of bearing branch is significantly improved; the bearing branch length of DP-1 (100 m^3^ hm^-2^), DP-2 (135 m^3^ hm^-2^) and DP-3 (180 m^3^ hm^-2^) are respectively 17.0 cm, 17.8 cm and 19.2 cm (Fig 1A). In 2012 the bearing branch length of DP-3 is grow the fastest reaching 22.0 cm; it is 47.7% higher than C; and it is significantly different from other treatments (*P*<0.05). Then the bearing branch of DP-2 is reached 19.5 cm which is 30.9% higher than C; and significantly different from C (*P*<0.05). But it is no significant difference with DP-4 and DP-5 (Fig 1D). In addition, in the same irrigation system, the growth rate of bearing branch is different in different years. The general result is the growth rate of bearing branch increases with the increase of years, while the bearing branch length of C fluctuates greatly (Fig 1A–1D).

### Effects of different irrigation system on reproductive growth of pear jujube

In 2009, the flower number of DP-1 is the least, which is 30009; followed by DP-3 treatment which is 32,084. The flower number of C is 37651; and it is significantly higher than that of other treatments. And in 2012, the flower number of C reached the maximum, and it is 39130. Different irrigation treatments significantly increase fruit number per plant, fruit setting rate and yield of pear jujube. In 2012, fruit number per plant of DP-3 is the largest which is 872, followed by DP-2 (864); and fruit number per plant of C has the least number which is 537.The trend of fruit setting rate of pear jujube in 2009–2012 is basically the same; it is DP-3> DP-2> DP-1> DP-4> DP-5>C. The fruit setting rates of DP-1, DP-2, DP-3, DP-4 and DP-5 reach the highest in 2012 and they are 2.34, 2.57, 2.76, 2.28 and 2.11, which are 0.97, 1.20, 1.39, 0.91 and 0.64 percentage points respectively higher than C, and both are significantly different from C (P<0.05). The yield of DP-3 from 2009 to 2010 is significantly different from other treatments (P<0.05), and increased by 7.5% and 7.9% respectively compared with C; in 2012, the highest yield of pear jujube is treated with DP-3 to be 16772.8 kg hm^-2^. Next, the DP-2 treatment is 16658.2 kg hm^-2^, which is 13.2% and 12.4% higher than C, respectively; and the difference is significant (P<0.05) (Table 3).

**Table 3:** Effect of jujube reproductive growth in different irrigation system from 2009 to 2012.

Year	Treatment	flowers	Number of fruits	Fruiting set (%)	yield (kg hm^-2^)
**2009**	DP-1	30 009 f	615 c	2.05 bc	13 091.9 bc
	DP-2	33 078 d	741 a	2.24 ab	13 229.1 b
	DP-3	32 084 e	760 a	2.37 a	13419.9 a
	DP-4	33 688 c	664 b	1.97 bc	13 152.2 bc
	DP-5	34 809 b	668 b	1.92 c	12 988.8 c
	C	37 651 a	459 d	1.22 d	12 483.9 d
**2010**	DP-1	30 062 f	643 c	2.14 bc	12 196.6 cd
	DP-2	33 541 d	775 a	2.31 ab	12 341.4 b
	DP-3	31 237 e	762 a	2.44 a	12 550.2 a
	DP-4	33 841 c	697 b	2.06 cd	12 264.5 bc
	DP-5	34 966 b	678 b	1.94 d	12 093.0 d
	C	38 874 a	501 d	1.29 e	11 628.6 e
**2011**	DP-1	30 200 f	661 d	2.19 b	14 446.4 bc
	DP-2	33 279 d	792 a	2.38 a	14 549.2 ab
	DP-3	31 275 e	788 a	2.52 a	14 711.2 a
	DP-4	33 779 c	740 b	2.19 b	14 487.1 b
	DP-5	35 000 b	711 c	2.03 b	14 308.9 c
	C	38 842 a	513 e	1.32 c	13 782.4 d
**2012**	DP-1	30 488 f	713 d	2.34 c	16 514.5 bc
	DP-2	33 567 d	864 a	2.57 b	16 658.2 ab
	DP-3	31 563 e	872 a	2.76 a	16 772.8 a
	DP-4	34 067 c	777 b	2.28 cd	16 559.4 bc
	DP-5	35 288 b	745 c	2.11 d	16 402.7 c
	C	39 130 a	537 e	1.37 e	14 822.3 d

Notes: Different letters in the same year indicate significant differences (P < 0.05) within the same column. DP-1: irrigation quota of 100 m^3^ hm^-2^ and irrigation 4 times; DP-2: irrigation quota of 135 m^3^ hm^-2^ and irrigation 4 times; DP-3: irrigation quota of 180 m^3^ hm^-2^ and irrigation 4 times; DP-4: irrigation quota of 135 m^3^ hm^-2^ and irrigation 3 times; DP-5: irrigation quota of 135 m^3^ hm^-2^ and irrigation 2 times; C: no irrigation.

### Effects of different irrigation system on soil moisture content of jujube forest

**Fig 2:**
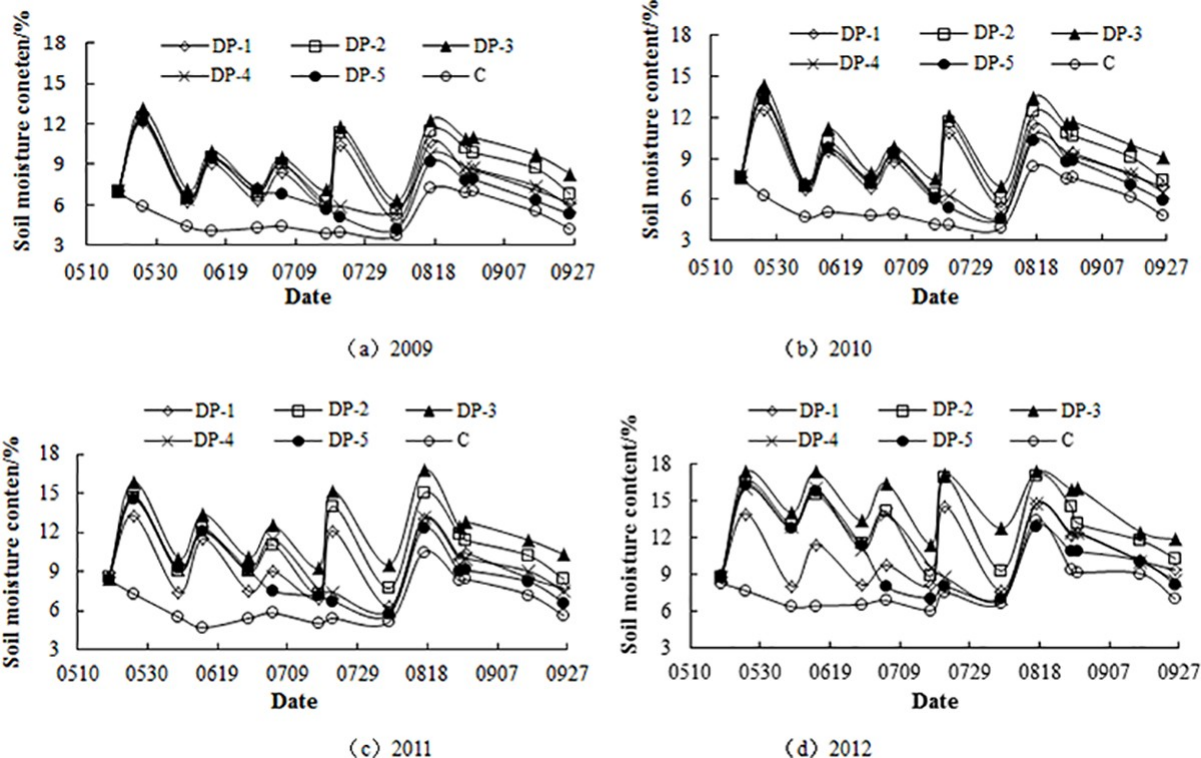
Dynamic change of soil moisture content in different treatments from 2009 to 2012. DP-1: irrigation quota of 100 m^3^ hm^-2^ and irrigation 4 times; DP-2: irrigation quota of 135 m^3^ hm^-2^ and irrigation 4 times; DP-3: irrigation quota of 180 m^3^ hm^-2^ and irrigation 4 times; DP-4: irrigation quota of 135 m^3^ hm^-2^ and irrigation 3 times; DP-5: irrigation quota of 135 m^3^ hm^-2^ and irrigation 2 times; C: no irrigation.

The different irrigation systems in 2009 are insignificant. As the years increase, the differences irrigation systems gradually increase. The soil moisture content increases with increasing of irrigation quota. The soil moisture content of DP-3 is higher than other treatments; and the soil moisture content of different drip irrigation is significantly higher than C for continuous four years. In early stage of growth period, due to the same irrigation date and irrigation quota (135 m^3^ hm^-2^) of DP-2, DP-4 and DP-5, the soil moisture content is not significantly different; in middle stage, due to the reduction of irrigation times of DP-4 and DP- 5, the soil moisture content decrease significantly; in the later stage, due to the influence of rainfall, the gap of soil moisture content (irrigated and non-irrigated) become smaller, and the soil moisture content of DP-2, DP-4 and DP-5 treatments is similar. During the whole growth period, the average soil moisture content of DP-1, DP-2, DP-3, DP-4 and DP-5 are 2.85, 4.08, 5.09, 2.67 and 2.42 percentage points respectively higher than C (Fig 2A–2D).

### Effects of different irrigation system on water consumption and crop coefficient of pear jujube

In 2009–2012, the water consumption of DP-3 is the highest in different growth period. The total water consumption of DP-3 from 2009 to 2012 are 381.6, 375.4, 422.6 and 403.1 mm, respectively; they are significantly higher than other treatments (*P*<0.05); while the water consumption of C is significantly lower than other irrigation treatments for four consecutive years. In the whole growth period, the water consumption during the fruit expansion period is the largest, followed by the flowering fruit setting period, with an average of about 38% and 28% of the total water consumption; and the minimum water consumption during the budding leaf period, which is about 13% in the total water consumption. When the irrigation times are the same, the water consumption of jujube trees increases with the increase of irrigation quota; when the irrigation quota is the same, the water consumption increases with the increase of irrigation times. The water consumption increases with the increase of irrigation amount every year. The crop coefficient of pear jujube continued to increase with the extension of the growth period, reaching the maximum during the fruit expansion period, and then decreased. The crop coefficient of DP-3 is the highest at different growth period in each year; from 2009 to 2012, the crop coefficients during the fruit expansion period are 1.16, 1.14, 1.29 and 1.25 (Table 4).

**Table 4:** Water consumption and crop coefficient of jujube trees treated differently from 2009 to 2012.

year	Treatment	Budding leaf period		flowering fruit setting period		expansion period		maturity period		Total water consumption (mm)
		water consumption (mm)	Kc	water consumption (mm)	Kc	water consumption (mm)	Kc	water consumption (mm)	Kc	
**2009**	DP-1	38.3	0.42	65.6	0.70	144.5	1.04	68.1	0.88	316.5 c
	DP-2	47.8	0.52	76.5	0.82	156.6	1.13	77.3	1.00	358.2 b
	DP-3	54.3	0.60	82.4	0.88	161.6	1.16	83.3	1.08	381.6 a
	DP-4	49.2	0.54	74.9	0.80	139.1	1.00	66.0	0.85	329.2 c
	DP-5	46.7	0.51	53.4	0.57	131.9	0.95	61.8	0.80	293.8 d
	C	31.5	0.35	32.2	0.34	103.6	0.75	44.7	0.58	212.0 e
**2010**	DP-1	27.5	0.38	97.4	0.85	116.3	0.99	55.3	0.78	296.5 c
	DP-2	33.2	0.46	105.6	0.92	121.3	1.03	65.8	0.93	325.9 b
	DP-3	44.1	0.61	118.6	1.04	134.4	1.14	78.3	1.10	375.4 a
	DP-4	31.5	0.44	108.2	0.94	97.8	0.83	42.3	0.60	279.8 c
	DP-5	35.3	0.49	92.6	0.81	89.2	0.76	37.6	0.53	254.7 d
	C	22.4	0.31	52.8	0.46	71.5	0.61	25.8	0.36	172.5 e
**2011**	DP-1	38.2	0.47	105.3	0.89	131.4	1.09	75.7	1.11	350.6 c
	DP-2	44.3	0.55	110.1	0.93	144.6	1.20	83.5	1.22	382.5 b
	DP-3	53.5	0.66	119.7	1.01	155.2	1.29	94.2	1.38	422.6 a
	DP-4	46.8	0.58	109.4	0.92	115.8	0.96	66.2	0.97	338.2 c
	DP-5	45.1	0.55	83.7	0.71	89.4	0.74	42.8	0.63	261.0 d
	C	26.3	0.32	41.5	0.35	87.9	0.73	23.4	0.34	179.1 e
**2012**	DP-1	30.0	0.37	94.6	0.87	111.7	1	93.9	1.02	330.2 c
	DP-2	37.1	0.46	105.4	0.97	126.2	1.13	98.5	1.07	367.3 b
	DP-3	46.5	0.57	112.0	1.03	139.7	1.25	105.0	1.14	403.1 a
	DP-4	34.4	0.42	89.1	0.82	108.4	0.97	80.1	0.87	312.0 c
	DP-5	40.3	0.5	75.0	0.69	95.0	0.85	69.1	0.75	279.3 d
	C	27.4	0.34	50.0	0.46	79.3	0.71	56.2	0.61	212.9 e

Notes: Different letters in the same year indicate significant differences (P < 0.05) within the last column. Kc: crop coefficient; DP-1: irrigation quota of 100 m^3^ hm^-2^ and irrigation 4 times; DP-2: irrigation quota of 135 m^3^ hm^-2^ and irrigation 4 times; DP-3: irrigation quota of 180 m^3^ hm^-2^ and irrigation 4 times; DP-4: irrigation quota of 135 m^3^ hm^-2^ and irrigation 3 times; DP-5: irrigation quota of 135 m^3^ hm^-2^ and irrigation 2 times; C: no irrigation.

### Effect of different irrigation system on yield and water use efficiency of pear jujube

Table 5 shows the water use efficiency of different irrigation systems in 2009–2012 as the increase in output per unit of irrigation. The yield of DP-3 in different irrigation systems from 2009 to 2012 is higher than other treatments, with yields of 13419.9, 12550.2, 14711.2 and 16772.8 kg hm^-2^, respectively; while DP-5 treatment is the lowest in irrigation treatment, with yields of 12988.8, 1093.0, 14308.9 and 16402.7 kg hm^-2^, respectively; DP-3 and DP-5 are significantly higher than C. Within four years, the water use efficiency of pear jujube in different irrigation systems is consistent, which is represented by DP-5>DP-4>DP-1>DP-2>DP-3. The water use efficiency of pear jujube shows that the low irrigation quota is better than the high irrigation quota. In 2012, the WUE of DP-1, DP-2 and DP-3 are 1.73, 1.55 and 1.32, respectively. The water use efficiency of DP-3 is the lowest for four consecutive years, which are 1.30, 1.28, 1.29 and 1.32, respectively; the water use efficiency of DP-5 is the highest for four consecutive years, which are 1.87, 1.72, 1.95 and 2.15, respectively. The high-irrigation treatment of pear jujube has the highest yield, but the water use efficiency is the lowest among the drip irrigation treatments; while the DP-5 treatment has the lowest total irrigation rate, and the yield of pear jujube is the lowest among the drip irrigation treatments, but the water use efficiency is the highest. The average irrigation water production efficiency of DP-5 is 1.92 kg m^-3^.

**Table 5:** Yield of jujube trees and irrigation water production efficiency in different irrigation system from 2009 to 2012.

Treatment	2009		2010		2011		2012	
	Yield (kg hm^-2^)	WUE (kg m^-3^)	Yield (kg hm^-2^)	WUE (kg m^-3^)	Yield (kg hm^-2^)	WUE (kg m^-3^)	Yield (kg hm^-2^)	WUE (kg m^-3^)
**DP-1**	13 091.9 bc	1.52 c	12 196.6 cd	1.42 c	14 446.4 bc	1.66 c	16 514.5 bc	1.73 c
**DP-2**	13 229.1 b	1.38 d	12 341.4 b	1.32 d	14 549.2 ab	1.42 d	16 658.2 ab	1.55 d
**DP-3**	13 419.9 a	1.30 e	12 550.2 a	1.28 d	14 711.2 a	1.29 e	16 772.8 a	1.32 e
**DP-4**	13 152.2 bc	1.65 b	12 264.5 bc	1.57 b	14 487.1 b	1.74 b	16 559.4 bc	1.82 b
**DP-5**	12 988.8 c	1.87 a	12 093.0 d	1.72 a	14 308.9 c	1.95 a	16 402.7 c	2.15 a
**C**	12 483.9 d	/	11 628.6 e	/	13 782.4 d	/	15 822.3 d	/

Notes: Different letters in the same year indicate significant differences (P < 0.05) within the same column. *WUE*: water use efficiency; DP-1: irrigation quota of 100 m^3^ hm^-2^ and irrigation 4 times; DP-2: irrigation quota of 135 m^3^ hm^-2^ and irrigation 4 times; DP-3: irrigation quota of 180 m^3^ hm^-2^ and irrigation 4 times; DP-4: irrigation quota of 135 m^3^ hm^-2^ and irrigation 3 times; DP-5: irrigation quota of 135 m^3^ hm^-2^ and irrigation 2 times; C: no irrigation.

### Determination of irrigation system for pear jujube forest

The rainfall data of the study area between 1956 and 2012 is obtained through the Mizhi weather station; and find that 2009–2012 are all multi-water years. The water deficit in each growth period is obtained from the rainfall and water demand (Table 6) during the growth period of pear jujube. Due to the rainfall maldistribution during the year in the Loess Plateau of northern Shaanxi, the water deficit of jujube is mainly concentrated in the budding leaf period, flowering and fruiting period; and the water deficit of flowering and fruiting period is the largest. Water deficit occurs when the rainfall during the fruit growing period is small. There is abundant rainfall during the ripening period of the fruit, and there is generally no shortage. Therefore, the multi-water year is mainly supplemented by the budding leaf period, flowering and fruiting period; and the expansion period should be supplementary irrigation according to the rainfall situation. Combining the different irrigation on the growth, yield, water use efficiency and water consumption of jujube trees, the cost-effective irrigation system and high-yield production irrigation system in multi-water year is determined as shown in Table 7; taking into account the interests of producers and the lack of water resources in the Loess Plateau in northern Shaanxi. Cost-effective is suggested in this research. Compared with the high-yield type, its irrigation amount is reduced by 62.6%, water use efficiency is increased by 62.8%, and production is only reduced by 2.21%. Medina water year and multi-water year use cost-effective irrigation (Table 7).

**Table 6:** Water deficit of jujube trees during each growth period from 2009 to 2012 mm.

year	Budding leaf period			Flowering fruit setting period			expansion period			maturity			Total deficit
	Rainfall	Water demand	Deficit	Rainfall	Water demand	Deficit	Rainfall	Water demand	Deficit	Rainfall	Water demand	Deficit	
**2009**	34.52	44.68	10.16	5.46	65.76	60.30	182.82	141.72	0	73.80	67.33	0	70.46
**2010**	12.44	31.12	18.68	29.53	96.25	66.72	102.70	109.06	6.36	70.60	51.40	0	91.76
**2011**	24.98	41.72	16.74	33.40	96.62	63.22	131.79	123.77	0	68.10	65.70	0	79.96
**2012**	26.57	37.85	11.28	60.44	108.26	47.82	97.39	107.46	10.07	92.50	78.40	0	69.17

**Table 7:** Drip irrigation jujube irrigation system.

years	Type	Irrigation quota (m^3^ hm^-2^)	Irrigation quota (m^3^ hm^-2^)	Irrigation times			
				Budding leaf period	flowering fruit setting period	expansion period	maturity period
**Multi-water year**	High yield	720	180	1	2	1	/
	*Cost-effective	270	135	1	1	/	/
**Medina water year**		675	135	2	2	1	/
**Drought year**		810	135	2	2	2	/

Note: *——Recommended irrigation system for the Multi-water year

## Discussions

This study found that different drip irrigation system significantly affects the growth, yield and water use of pear jujube in northern Shaanxi. Sun et al. [19] found that the irrigation method is the same, the growth of bearing branch increases with the increase of irrigation amount. This is consistent with the results of this study. With the increase of irrigation amount, the bearing branch length is significantly increased; Excessive water supply will promote the excessive development of branches, the competition between vegetative organs and reproductive organs will increase, and the workload of summer shears will increase. Wang et al. [20] and Ai et al. [21] conducted a pilot study on fruit trees and found that regulated deficit irrigation can effectively reduce the amount of shoot growth without affecting the economic benefits of crops. Therefore, in the research, the pursuit of high yield and excessive irrigation should not be excessively applied which not only helps to alleviate the shortage of water resources, but also reduces the amount of pruning and maximizes economic benefits.

In this study, when the irrigation quota is 135 m^3^·hm^-2^, the yield of pear jujube increases with the increase of drip irrigation times. This is consistent with another researcher's found [22–24]. Studies have shown that crop yield is positively correlated with irrigation quota; and proper increase irrigation quota is benefit to increase crop yield [25–26]. This is basically consistent with the results of this study. Different irrigation system has increased the yield of pear jujube, and yield increases with the increase of irrigation quota. Mainly because the water is sufficient to increase the water absorption and utilization of plant roots, promote physiological growth, increase the accumulation of dry matter, and ultimately increase the yield of pear jujube. The study found that the flower number of pear jujube is negatively correlated with the yield. The flower number of C in different years is significantly higher than irrigation treatments. This may be because the flower number and fruit setting rate are investigated in the early stage of flowering fruit setting period; and non-irrigated pear jujube is quickly flowering and fruit setting in this period, but the accumulation of dry matter is not enough, resulting in serious flower and fruit drop; and finally the number of fruit set is less, the fruit setting rate is low, and the total yield decreased. The yield of the same treatment shows an increase trend from 2009 to 2012. This may be caused by long-term irrigation, which improves the water content of the soil of the pear jujube forest in the northern Shaanxi, and is conducive to the growth of jujube trees.

Appropriate irrigation can provide plants with the water needed for growth in a timely, effectively improve the soil environment, and achieve the purpose of improving production, quality and water use efficiency. This study found that different drip irrigation systems effectively improve the soil moisture content of pear jujube forest. This is basically consistent with the research results of Li et al. [27]. This study shows that the water consumption of pear jujube increases with the increase of irrigation quota and irrigation times. And it is consistent with the research results of Zhang et al. [28] and Zheng et al. [29]; the water consumption during the growth period is greatly affected by soil moisture. Irrigation can effectively replenish the soil water deficit caused by plant growth. It is greater of irrigation amount and the moister the soil; and the water consumption of the plant is greater. The crop coefficient is influenced by multiple factors such as meteorology and irrigation in each year which are differences between years. The trend of crop coefficient is consistent in the whole growth period of pear jujube, and the crop coefficient of pear jujube is continuing to increase then it declines until the fruit expansion period. This may be due to the physiological and reproductive growth of pear jujube requiring a large amount of water supply, and the water consumption of pear jujube is increasing, so that the crop coefficient shows an increasing trend. The crop coefficient and the water consumption of pear jujube are basically consistent.

Nie et al. [30] show that under the insufficient rainfall, the WUE of drip irrigation system with reduced irrigation amount is significantly higher than flood irrigation. Çetin et al. [31] have shown that subsurface drip irrigation can effectively reduce water consumption and improve water use efficiency. This is basically consistent with the results of this study. Deficit treatment is carried out at a certain growth period to effectively improve the water use efficiency of pear jujube. Studies have shown that the reduction of irrigation amount in specific growth period has little effect on the yield of kiwifruit, and the water use efficiency has been greatly improved [32–33]. Cui et al. [34], Zhang et al. [35] and Zhang et al. [36] have shown that yields increase first and then decrease, while water use efficiency decreases significantly with a certain amount of irrigation. In this study, the yield of pear jujube increase with the increasing of irrigation quota. When the yield is the highest, the water use efficiency is not necessarily the largest; and the effect of reducing irrigation on the yield of pear jujube is not significant. Therefore, when the yield of pear jujube is guaranteed, moderate water shortage is beneficial to improve water use efficiency, and the high water use efficiency value often appears medium water supply [37–38]; but there are differences in other research results [26], which may be related to different subjects, irrigation frequency, irrigation quota setting, climate and site conditions in the test area. Liu et al. [39] showed that due to moderate water deficit, the vegetative growth of fruit tree is inhibited, and more assimilates are distributed to fruits, which explained the reason for the increase in yield and water use efficiency after moderate deficit treatment. Studies have shown that moderate fertilization under drought stress can increase the development level of crop roots and the ability to take in soil moisture, expand the space for water and nutrients, and improve water use efficiency [40–41]. The results are repeatedly verified in multi-water year after 2013; and the results were consistent with the test results. Therefore, the irrigation system can be promoted and applied throughout the northern Shaanxi region, and it can also be applied in other similar regions.

## Conclusion

The unique geomorphy and uneven rainfall in the loess hilly region of northern Shaanxi lead to a low degree of availability of natural precipitation, and the regional water resources are seriously deficient. Water-saving irrigation can effectively improve WUE and yield of pear jujube. Here, we have found that a cost-effective irrigation system can be used in multi-water year; it is the DP-5 treatment (irrigation quota is 135 m^3^ hm^-2^, irrigation time is April 20 and May 17). This study is mainly carried out from the water content of pear jujube, and the irrigation system has been applied for many years in northern Shaanxi, and the output of orchard has reached the expected target. In the future study, it is necessary to further research the high-efficiency irrigation system under the coupling of water and fertilizer and the quality of pear jujube.

## Supporting information

S1 Table Bearing branch length from 2009 to 2012. (XLSX)

S2 Table Dynamic change of soil moisture content in different treatments from 2009 to 2012. (XLSX)
